# Plant growth promotion and differential expression of defense genes in chilli pepper against *Colletotrichum truncatum* induced by *Trichoderma asperellum* and* T*.* harzianum*

**DOI:** 10.1186/s12866-023-02789-x

**Published:** 2023-03-03

**Authors:** Mukesh Yadav, Kumari Divyanshu, Manish Kumar Dubey, Ashutosh Rai, Sunil Kumar, Yashoda Nandan Tripathi, Vaishali Shukla, Ram Sanmukh Upadhyay

**Affiliations:** 1 Laboratory of Mycopathology and Microbial Technology, Centre of Advanced Study in Botany, Institute of Science, Banaras Hindu University, Varanasi, 221005 Uttar Pradesh India; 2 Kutir Post Graduate College Chakkey, Jaunpur, 222146 Uttar Pradesh India; 3 Department of Biosciences, School of Basic & Applied Sciences, Galgotias University, Greater Noida, 203201 Uttar Pradesh India; 4 Department of Biochemistry, College of Horticulture, Banda University of Agriculture and Technology, Banda, 210001 Uttar Pradesh India; 5 Government Post Graduate College, Obra, Sonbhadra, Uttar Pradesh, 231219 India

**Keywords:** Bio-priming, *Capsicum annuum*, Defense related genes, Electron microscopy, Growth promotion, Lignification, Real-time PCR

## Abstract

**Background:**

*Trichoderma* *asperellum* and *T*. *harzianum* were assessed in this study as a potential biological control against *Colletotrichum truncatum*. *C. truncatum* is a hemibiotrophic fungus that causes anthracnose disease in chilli thereby affecting plant growth and fruit yield. Scanning electron microscope (SEM) technique showed the beneficial interaction between chilli root-*Trichoderma* spp. inducing the plant growth promotion, mechanical barrier, and defense network under *C*. *truncatum* challenged conditions.

**Methods:**

Seeds bio-primed with *T*. *asperellum*, *T*. *harzianum*, and *T. asperellum* + *T. harzianum* promoted the plant growth parameters and strengthening of physical barrier via lignification on the wall of vascular tissues. Seed primed with bioagents were used for exploring the molecular mechanism of defense response in pepper against anthracnose to assess the temporal expression of six defense genes in the Surajmukhi variety of *Capsicum annuum*. QRT-PCR demonstrated induction of defense responsive genes in chilli pepper bioprimed with *Trichoderma* spp. such as plant defensin 1.2 (*CaPDF1.2*), superoxide dismutase (*SOD*), ascorbate peroxidase (*APx*), guaiacol peroxidase (*GPx*), pathogenesis related proteins PR-2 and PR-5.

**Results:**

The results showed that bioprimed seeds were assessed for *T*. *asperellum*,* T*. *harzianum*, and *T*. *asperellum* + *T*. *harzianum*-chilli root colonization interaction under in vivo conditions. The results of the scanning electron microscope revealed that *T*. *asperellum*, *T*. *harzianum* and *T*. *asperellum* + *T*. *harzianum* interact with chilli roots directly via the development of plant-*Trichoderma* interaction system. Seeds bio-primed with bioagents promoted the plant growth parameters, fresh and dry weight of shoot and root, plant height, leaf area index, number of leaves, stem diameter and strengthening of physical barrier via lignification on the wall of vascular tissues and expression of six defense related genes in pepper against anthracnose.

**Conclusions:**

Application of *T*. *asperellum* and *T*. *harzianum* and in combination of treatments enhanced the plant growth. Further, as seeds bioprimed with *T*. *asperellum*, *T*. *harzianum* and in combination with treatment of *T*. *asperellum* + *T*. *harzianum* induced the strengthening of the cell wall by lignification and expression of six defense related genes *CaPDF1.2, SOD, APx, GPx, PR-2* and *PR-5* in pepper against *C*. *truncatum*. Our study contributed for better disease management through biopriming with *T*. *asperellum*, *T*. *harzianum* and *T*. *asperellum* + *T*. *harzianum*. The biopriming possess enormous potential to promote plant growth, modulate the physical barrier, and induced the defense related genes in chilli pepper against anthracnose.

## Background

Chilli (*Capsicum annuum* L.) member of the family Solanaceae, is an important crop cultivated in tropical and subtropical countries including India. It is regarded as an essential spice used in daily cuisines across the world. Chilli peppers are abundant in capsaicin, capsidiol, vitamin A, C and E, folic acid, fibres, capsochrome, and protein. According to the report of [1]**,** India is the world’s largest producer, consumer and exporter of red pepper. In India, the total production of green and red chillies was estimated 0.0679 million tonnes and 1.389 million tonnes, cultivated in an area of 0.8 million hectares [2]. However, pepper production has been affected by biotic and abiotic stresses such as *Colletotrichum* spp., *Phytophthora capsici*, *Pythium* spp., *Fusarium solani* var. *capsici*, *Rhizoctonia solani*, *Xanthomonas campestris* pv. *vesicatoria*, *Ralstonia solanacearum*, leaf curl virus, *Meloidogyne incognita*, heat, drought, salinity and other abiotic factors [3, 4]. Anthracnose is the most devastating disease caused by *Colletotrichum* spp. namely, *C. truncatum*, *C*. *scovillei*, *C*. *acutatum*, *C*. *gloeosporioides* and *C*. *coccodes* belonging to the family Glomerellaceae of the phylum Ascomycota [5]. Among all other *Colletotrichum* spp., *C*. *truncatum* is the most prominent phytopathogen, associated with anthracnose in chilli, alone causing 50% yield loss worldwide [6]. Further, from the Indian perspective, the devastating fungus *C*. *truncatum*, alone caused 10 − 54.91% yield loss in India [7, 8].

Anthracnose symptoms include blacken spherical sunken injuries with concentrical rings of acervuli containing sickle shaped conidia causing fruit rot and reducing their quality and salability [9]. *C*. *truncatum* is primarily a seed- or air-borne pathogen that mainly infects fruits and leaves of chilli. Under favorable conditions, it spreads and causes an epidemic disease that leads to the reduction of crop yields worldwide [8]. For the control of this destructive pathogen, the application of fungicides is recommended. It reduces the anthracnose lesions progression and enhances crop yields, however, the increased accumulation of fungicides that affects the quality of chilli fruits remains a major constraint with this approach [10]. The heavy load of the chemical compound is unsustainable to agricultural fields and it also provides resistance to phytopathogens. However, since there are no resistant cultivars that have been evolved therefore, it is especially necessary to use biocontrol agents (BCAs) because they are profitable and environment-friendly, and can serve as a better alternative to chemical control [11].

Several studies have been commenced to reveal the plants primed with plant growth-promoting microorganisms emerging with the plant defense mechanism against the pathogen aggression by the activation of defense responsive genes [12, 13], production of free radicals leading to hypersensitive response, biosynthesis of pathogenesis-related (PR) protein, production of phytoalexins [8, 14], as well as lignification of vascular strands [15–17]. Among the plant growth promoting microbes, *Trichoderma longibrachiatum* and *Paenibacillus polymyxa* E681 are used as potent biocontrol agents in the improvement of plant immunity against pathogen attack [18, 19]. Transcription factor APETALA2/Ethylene Factor-domain directly governs the expression of *CaPDF1.2* (PLANT DEFENSIN1.2) in the way of pathogen infection in *Arabidopsis* [20]. For example, the expression of *GLU* (β-1, 3-glucanase gene) and *PIK1* (pathogen-induced kinase gene) defense genes have been increased in chilli treated with *T. harzianum* and* T. asperellum* under *C. truncatum* challenged condition [21]. Seed primed with *Bacillus cereus* AR156 induces systemic resistance in *Arabidopsis* against *Pseudomonas syringae* pv. tomato DC3000 via the activation of salicylic acid (SA) responsive genes *PR1*, *PR2*, *PR5*, and jasmonic acid (JA)/ethylene (ET) responsive gene *CaPDF1.2* [8, 13]. *Trichoderma asperelloides* PSU-P1 triggered the expression of defense responsive genes as well as promoted plant growth in *Arabidopsis thaliana* against the attack of phytopathogens [22]. The expressed defense responsive genes help in correlating the signaling pathways and subsequent effectors to reduce the effect of biological stresses [23]. However, still, there is a limited report on molecular defense system against anthracnose infection in chilli.

In the line with above findings, in the current study, we hypothesized that the activation of defense responsive genes in chilli pepper by *T. asperellum* and *T. harzianum* can exhibits biocontrol potential against anthracnose caused by *C*. *truncatum*. Therefore, this study sought to meet two key objectives: (I) evaluate the potential efficacy of *T. asperellum* and *T. harzianum* as biocontrol agents in plant growth promotion and strengthening of physical barrier (II) evaluation of *Trichoderma* spp. on the expression of defense related genes in red pepper against *C*. *truncatum* causing anthracnose in chilli. In this work, the attempt was made for the first time to study the differential expression of defense responsive genes in pepper treated with *T. asperellum* and *T. harzianum* upon *C. truncatum* challenged conditions*.*

## Materials and methods

### Plant materials and growth conditions

The susceptible chilli variety (*Capsicum annuum* cv. Surajmukhi) seeds were procured from the ICAR-IIVR, Varanasi, Uttar Pradesh, India. Surface sterilization of the obtained seeds was performed by using 1% NaOCl for 1–3 m with subsequent treatment with ethyl alcohol (70%) for the 30 s followed by 3X washing with sterilized distilled water and air drying for 2 h under laminar airflow. Sterile seeds were grown in mixed autoclaved soil prepared by combining clay and vermin compost in proportions of 3:1 (v/v) in a greenhouse condition with natural photoperiod at 27 ± 1°C. Finally, the plants were selected for testing the pathogenicity of *C. truncatum* in treated and untreated seeds and also for further experiments after attaining the fruiting stage i.e., when > 90% of fruits have typical fully ripe colour.

### Culture collection and growth conditions

*C*. *truncatum* reported in the previous study by Yadav et al. [2] was obtained from the Laboratory of Mycopathology and Microbial Technology, Department of Botany, Banaras Hindu University, Varanasi, Uttar Pradesh, India and used for further experiments on chilli pepper. The fungal cultures of *T. harzianum* (GenBank accession no-KR 856,210) and *T. asperellum* (GenBank accession no-KR 856,207) were acquired from the RY Roy Laboratory of Mycopathology, Department of Botany, Institute of Science, Banaras Hindu University, Varanasi, Uttar Pradesh, India.

Fungal growth was carried out in Petri dishes containing solidified potato dextrose agar (PDA) medium purchased from HiMedia Laboratories (Mumbai, India) supplemented with streptomycin sulphate (0.03 g/L) and chloramphenicol (0.05 g/L) at 27 ± 1°C for 7 days. Further, the isolates were maintained at 4°C on PDA medium for three months and revived thereafter for use.

### Preparation of fungal inocula

The inoculum suspension of *C. truncatum*, *T. harzianum* and *T. asperellum* was prepared using the method described previously by Yadav et al. [2]. The isolate of *C*. *truncatum* was grown on PDA medium for twenty days at 27°C. Subsequently, the Petri dish was flooded with 10 mL of sterilized distilled water, and the conidia were scratched using a sterile slide. The conidial suspension was filtered using a muslin cloth and afterwards, the filtered conidial inoculum was diluted with sterilized water to maintain 1 × 10^6^ conidia/mL by counting with a hemocytometer. Similarly, the preparation of an inoculum suspension of *T*. *asperellum* and *T*. *harzianum* was also carried out and maintained to a final concentration of 2 × 10^7^ conidia/mL.

### Seed priming with bioagents

The conidial suspension of *T*. *asperellum* and *T*. *harzianum* (2 × 10^7^ conidia/mL) were centrifuge for 15 min at 10,000 rpm. The pellets were suspended in 100 mL of sterile distilled water containing 1.5 g carboxymethyl cellulose (CMC) [15]. The susceptible variety of chilli seeds (cv. Surajmukhi) were surface sterilized with 1% sodium hypochlorite for 1–2 min followed by treatment with 70% of ethyl alcohol for 30 s and washed thrice with sterile distilled water and left for air drying in laminar flow for 2 h. The sterilized seeds were soaked in a conidial suspension of *T*. *asperellum*, *T*. *harzianum*, and *T. asperellum* + *T. harzianum* on a shaker for 12 h at 150 rpm. The soaked seeds were filtered and dried on sterile blotting paper in laminar airflow. The CMC-soaked seeds devoid of conidial suspension of bioagents served as control.

### Treatment of chilli seeds with *T. asperellum* and *T*. *harzianum* under *in-vitro* conditions

The bio-primed seeds were sown and grown in sowing pots in 15 × 10 cm^2^ containing mixed sterilized soil (1.5 kg) combining clay and vermin compost in proportions of 3:1 (v/v). Thereafter, soil drenching followed by foliar spraying operations were performed five times in the entire life cycle of the plant to treat the seedlings with *T. asperellum* and *T*. *harzianum*. Following the bio-agent treatments at the fruiting stage, the conidial suspension of *C*. *truncatum* was sprayed over the fruits and covered with sterilized plastic bags to retain the moisture for 96 h. The control was set up by using untreated and unchallenged chilli fruits sprayed with deionized water (DI) under similar conditions. The experiment comprised of the following five sets of treatments: *T*. *asperellum* + *T*. *harzianum* treated seeds, *T. asperellum* treated seeds, *T*. *harzianum* treated seeds upon pathogen-challenged and pathogen-inoculated samples. In contrast, the untreated and unchallenged seeds served as control. All the experiments were executed thrice in triplicates, moreover, for each treatment, three seedlings were maintained in three sowing pots.

### Scanning electron microscope (SEM) to study the In Vivo root colonization of plant growth promoting fungi (PGPF)

The thirty days old chilli seedlings treated with PGPF and untreated (control) seedlings were uprooted and washed with sterile distilled water. Afterwards, the root tissues were excised with a sterilized blade and fixed in 2.5% glutaraldehyde in 0.5 M sodium phosphate buffer (pH 6) and kept for 12 h at 4°C in a dry Petri plate. Finally, the dried root samples were tape-affixed and thereafter coated with Au [24]. The root samples were observed under scanning electron microscopy at 20 kV in ZEISS, model number EVO-18 (Germany) for fungal colonization [25]. The experiment consisted of three seedlings for each treatment and was repeated thrice. The fungi which colonized in chilli root in all the replicates were selected and used for further experiments.

### Assessment of seed treatment with bioagents on Plant growth parameters of chilli under greenhouse conditions

Plant growth promoting fungi treated and untreated control seeds were sown in mixed sterile soil (clay/vermi-compost, 3:1, v/v) in greenhouse conditions under 80% relative humidity with 14 h light and 10 h dark cycle at 27 ± 1°C for 75 days. After 30 and 60 days of sowing, chilli seedlings were uprooted delicately to record the plant growth parameters (leaf area index, plant height, root fresh and dry weight, shoot fresh and dry weight, the total number of leaves in a plant, leaves fresh and dry weight).

### Histochemical staining for detection of lignin deposition

The assessment of lignin deposition on vascular tissue (xylem) was performed using the method described by Saxena et al. [26]. The control and pre-treated chilli plants with bioagents were used for the observation of lignin deposition on cell-wall. After 30 and 60 days of treatment with bioagents, the second internodal region of the stem was selected. The transverse section of the stem of treated and untreated samples was made with hand-cut approx. 0.5 mm thickness with the help of a steel blade. The sections were fixed in 1% (w/v) phloroglucinol solution containing 50% HCl in the ratio of 3:1 and kept for 5 min. After that, the sections were mounted with glycerin on a glass side and covered with a coverslip. The stained tissue was observed under Olympus binocular microscope. The deposition of lignin in stem tissues was examined as red-pink color. The experiment was repeated thrice.

### Assessment of anthracnose resistance in chilli pepper after treatment with bioagents under *C*. *truncatum* challenged conditions

One hundred fourty days old plants containing fruits were surface sterilized with 1% sodium hypochlorite for 1–2 min followed by 70% of ethyl alcohol for 30 s and washed three times with sterile distilled water and left for drying. Surface sterilized fruits were inoculated with 10 µL of conidial suspension of *C*. *truncatum* (1 × 10^6^ conidia mL^−1^) at the proximal end with the help of a sterile syringe using the pin-prick inoculation method [27]. Inoculated chilli fruits were covered using autoclaved plastic bags for 96 h to retain humidity under greenhouse conditions with natural photoperiod at 27 ± 1°C. Fruits inoculated with sterile distilled water served as control. The chilli fruits were harvested at different time intervals 0-, 2- and 4-days post inoculation (dpi) of *C*. *truncatum* and stored at − 80°C for RNA isolation. The length of anthracnose lesion was recorded at 4 days post inoculation (dpi). The percent disease severity (PDS) was calculated by dividing lesion length by total fruit length. The percent disease severity was measured on a scale ranging from 0 to 4 [28]. The experiment was performed thrice in triplicate for each treatment.

### RNA extraction and complementary DNA (cDNA) synthesis

The total RNA was extracted from 0.1 g of chilli fruit frozen in liquid nitrogen using Trizol reagent (Invitrogen) along with DNAse I as per the manufacturer’s protocol [29]. The quality of extracted RNA was confirmed by 1% agarose gel containing 0.5 µg mL^−1^ ethidium bromide [30]. The evaluation of extracted RNA was further quantified and purified through Nanophotometer (Implen, CA, United States) at 230/260/280 nm absorption ratio. Further, the cDNA first-strand synthesis was carried out by using 1.0 µg of the extracted RNA with the iscript™ cDNA synthesis kit (Bio-Rad Laboratories, United States).

### Quantitative real time PCR analysis

The assessment of temporal expression of accumulated defense genes (*PDF-1, SOD, APx, GPx, PR-2* and *PR-5*) was done quantitatively in all four treatments. The first strand of cDNA was diluted to 50 ng/µL and used as a template for qRT-PCR. qRT-PCR reactions were carried out using specific primers sequence designed from six defence-related genes (Table 1). qRT-PCR was performed thrice in triplicates for each independent treatment by using SsoFast^TM^EvaGreen^R^ Supermix detection chemistry (Bio-Rad) with an iQ5 thermocycler (BioRad Laboratories, United States). The process was executed using SYBR Green fluorescence dye (Qiagen, United States) and analyzed by employing iQ-SYBR Green Supermix (Bio-Rad, CA, United States) on iQ5 thermocycler (Bio-Rad, CA, United States) with iQ5 Optical System Software version 2.0 (Bio-Rad, CA, United States). For qRT-PCR reactions, the final volume of 20 µL was set and the reaction mixture comprised of 2 µL of cDNA template (20 ng), 10 µL of 2 X SsoFast^TM^EvaGreen^R^ Supermix, 1µL each of forward and reverse primers (0.2 µM) and 1 µL of nuclease-free water. Further, the reactions were subjected to an initial step of 95°C for 10 m followed by 45 cycles of 95 °C for 15 s, annealing at 60°C for the 30 s and extension at 72°C for 30 s. Relative defense gene expression was analyzed by using the 2^−∆∆Ct^ method according to the protocol as previously described by Livak and Schmittgen [31].

**Table 1:** Primer sequence and accession number

Genes	Primer sequence	Accession number
Plant Defensin 1.2 (*CaPDF 1.2*)	F 5´-GCTTCCATCATCACCCTTATCT-3́ R 5´-CATGTCCCACTTGGCTTCT-3́	**NM_123809.4**
Superoxide Dismutase (*CaSOD*)	F 5´-CCTTCGCCGGAATTCTAACA-3́ R 5´-GCAGGTTCTAAGGCTCCATAAT-3́	**XM_016717949.1**
Ascorbate Peroxidase (*CaAPx1*)	F 5´-GAAAGTGAGGGCCTACTGAAA-3́ R 5´-CCTTAGCATACAGCTCGACATAG-3́	**XM_016684466.1**
Guaiacol Peroxidase (*CaPOD*)	F 5´-GCTAGGGACTCTGTTGTCATTC-3́ R 5´-GGAGGAGGAATGCTGCTATTG-3́	**XM_016717947.1**
Endo-1,3-β-Glucanase (*CaPR-2*)	F 5´-GCGGACATGGCTCTGTATTAG-3́ R 5´-CATAAACGTCGGAGACGAAGAG-3́	**XM_016689727.1**
Pathogenesis-Related Protein 5 (*CaPR-5*)	F 5´-GGTGATTGTGGCTCATCTCTAA-3́ R 5´-CCATCAACAAGGCTAACATCATAAA -3́	**XM_016716165.1**

### Statistical analysis

The data were examined using the statistical software SPSS ver. 16.0. The experiments were performed thrice in triplicates of each treatment, and one-way analysis of variance (ANOVA) was used for the results comparison, analysis and examination. Moreover, Duncan’s multiple range test at p values ≤ 0.05 was used to express the significant differences in the means of each data.

## Results

### SEM observation of root colonization of PGPF

**Fig. 1:**
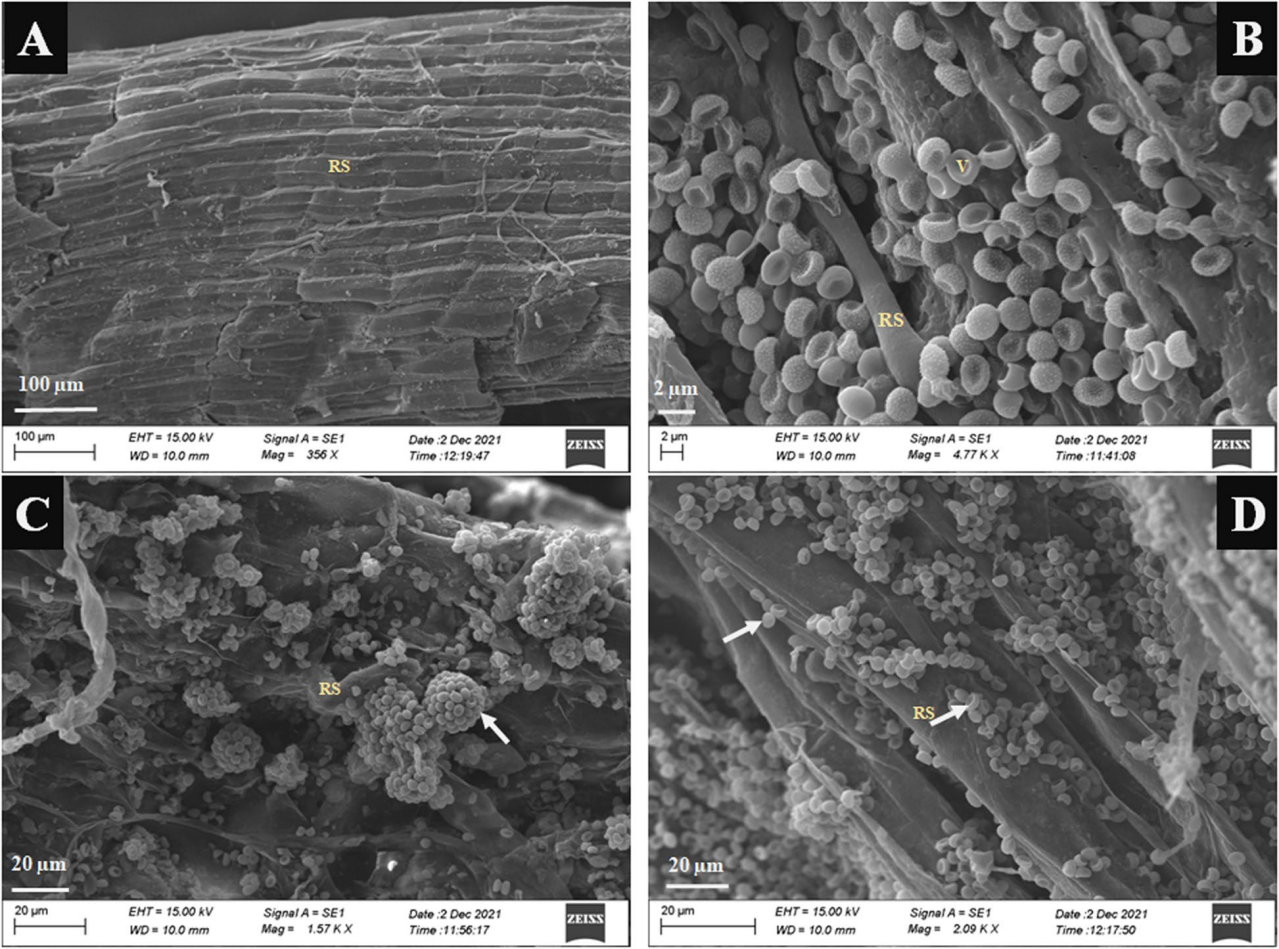
SEM images of chilli root colonized by *Trichderma* spp. **A** Uncolonized roots, **B** Colonized by *T. asperellum*, **C** Colonized by *T. harzianum*, **D** Colonized by *T. asperellum* + *T. harzianum*. The arrows indicate conidia of *Trichoderma* spp.: valleys (V), root surface (RS)

The bioprimed seeds were assessed for *T*. *asperellum*,* T*. *harzianum*, *T*. *asperellum* + *T*. *harzianum*-chilli root colonization interaction under in vivo conditions. The comparison between untreated (control) roots (Fig. 1 A), and treated with PGPF on the chilli root surface was observed through a scanning electron microscope (Fig. 1 B–D). The results of the scanning electron microscope revealed that *T*. *asperellum*, *T*. *harzianum*, and *T*. *asperellum* + *T*. *harzianum* interact with chilli roots directly via the development of plant-*Trichoderma* interaction system.

### Assessment of bioprimed seeds on plant growth parameters of chilli under glasshouse conditions

**Fig. 2:**
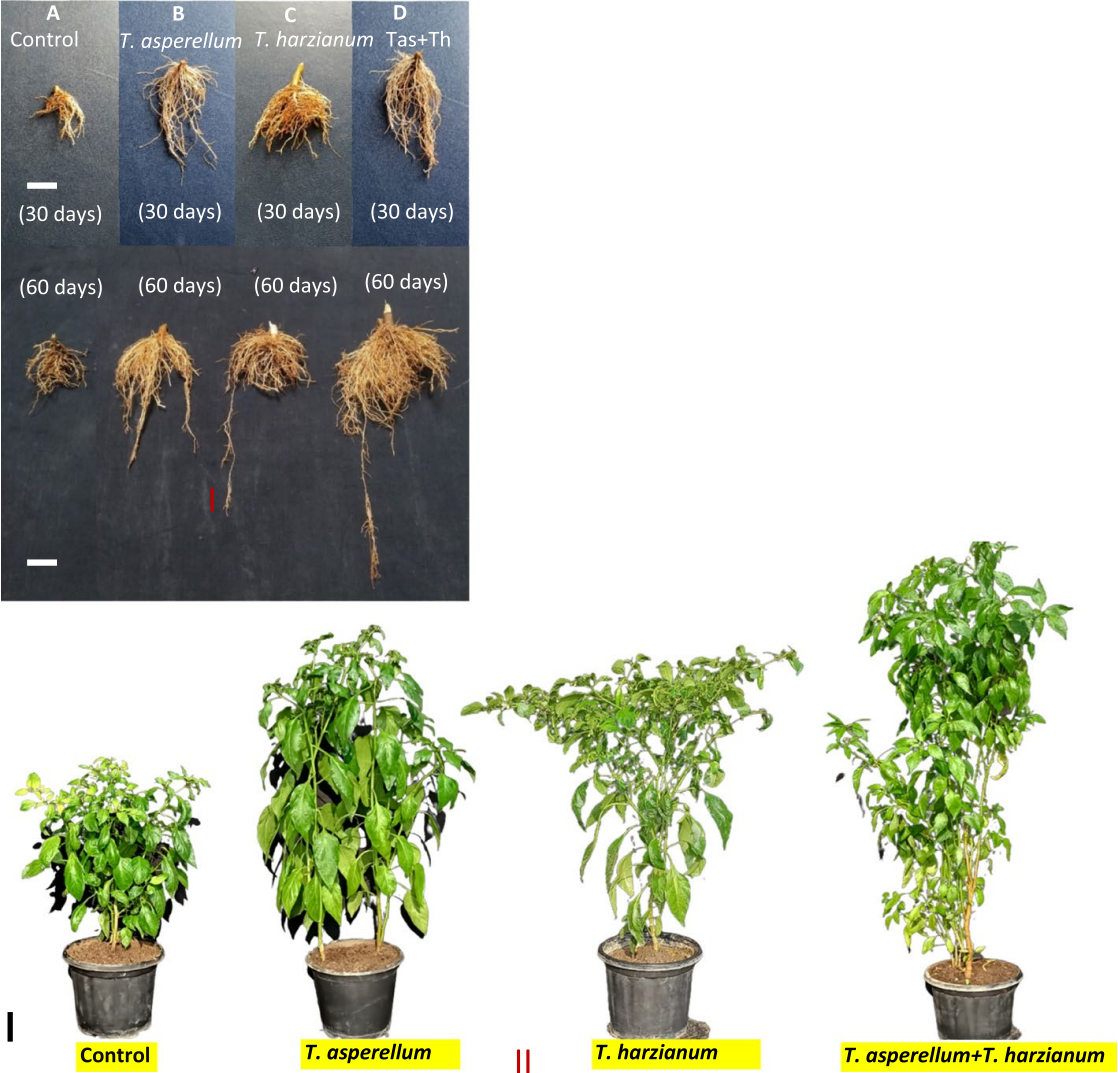
(I) Morphological growth characteristics of *T*. *asperellum*, *T*. *harzianum* and *T. asperellum* + *T. harzianum* bioprimed and untreated chilli plants at different intervals of 30 and 60 days. Seeds bioprimed samples were found to have increased the root length, profuse growth and thickness. **A** Image showing the unprimed (control) plant after 30 and 60 days, **B** *T*. *asperellum* bioprimed seeds after 30 and 60 days, **C** *T. harzianum* bioprimed seeds after 30 and 60 days, **D** *T. asperellum* + *T. harzianum* bioprimed seeds after 30 and 60 days. (II) Bioprimed seeds with *T*. *asperellum*, *T*. *harzianum* and *T. asperellum* + *T. harzianum* showed their growth parameters, increased in height, number and size of leaves, branches, flowering buds, stem diameter of plants. **A** Unprimed (control) plants, **B** Seeds were primed *T*. *asperellum,* **C** Seeds were primed with *T*. *harzianum*, and **D** Seeds were primed with *T. asperellum* + *T*. *harzianum*. Scale bar represents 10 cm

**Fig. 3:**
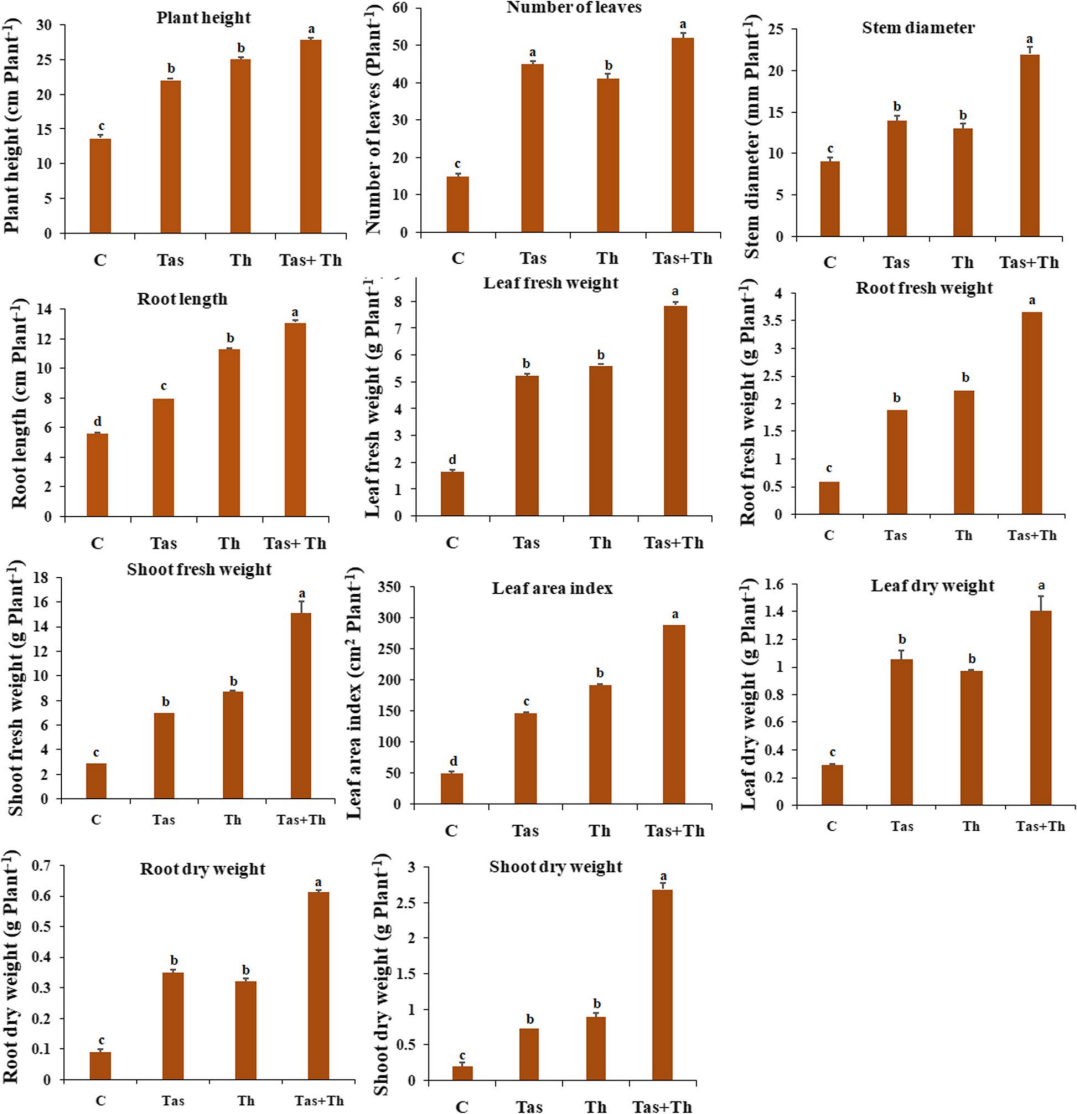
Effect of *T*. *asperellum*, *T*. *harzianum* and *T. asperellum* + *T. harzianum* bioprimed seeds on plant growth parameters of chilli under glasshouse conditions at 30 days. Each data indicated the mean of triplicates value, and the vertical bars give out the same alphabetical letters are not significantly different (*p* ≤ 0.05) using Duncan’s multiple range test. The bar denotes the SE of the mean

**Fig. 4:**
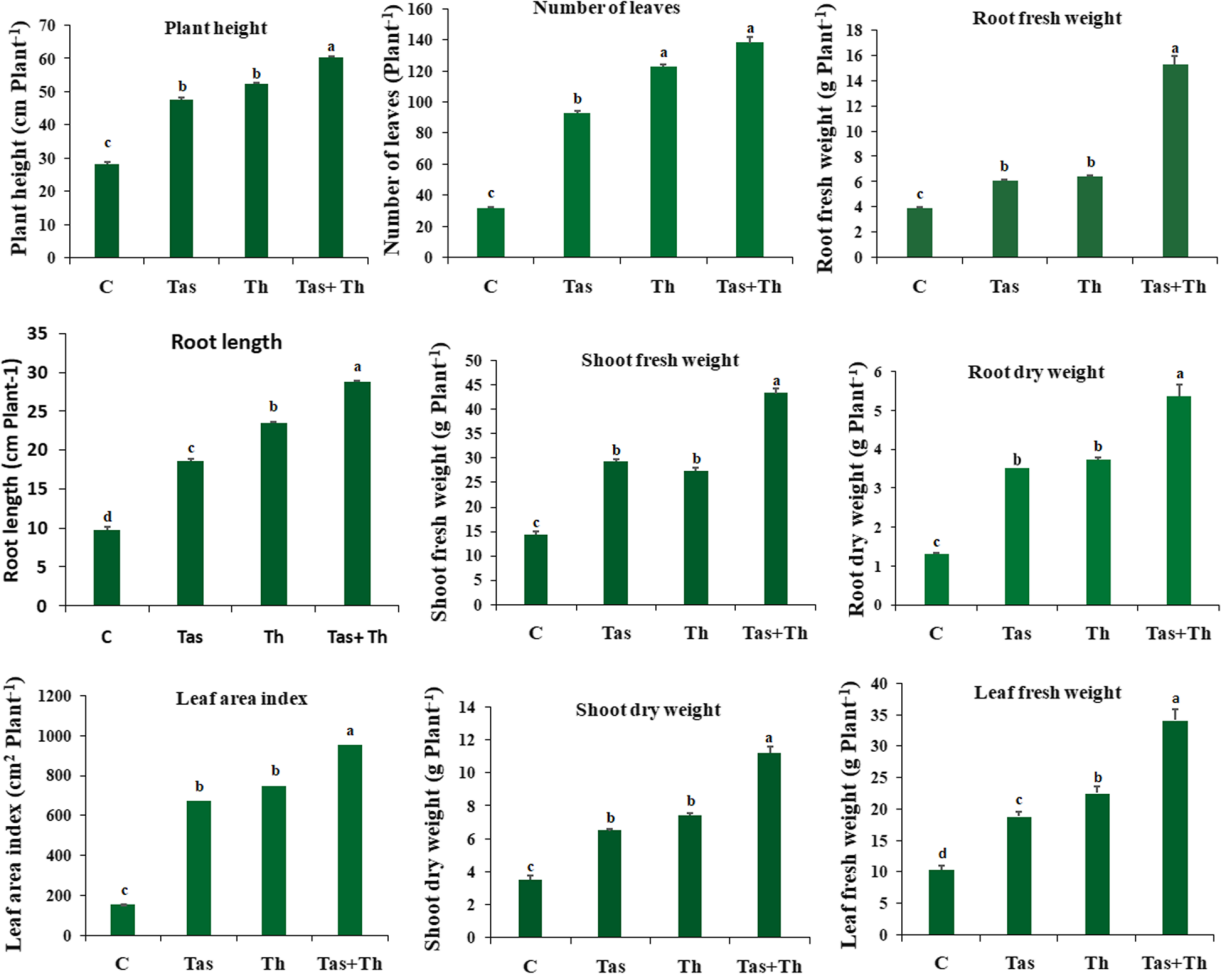
Effect of *T*. *asperellum*, *T*. *harzianum* and *T. asperellum* + *T. harzianum* bioprimed seeds on plant growth parameters of chilli under glasshouse conditions at 60 days. Each data indicated the mean of triplicates value, and the vertical bars give out the same alphabetical letters are not significantly different (*p* ≤ 0.05) using Duncan’s multiple range test. The bar denotes the SE of the mean

Seeds bio-primed with *T. asperellum*, *T. harzianum* and T. *asperellum* + *T. harzianum* were evaluated for their effect on plant growth promoting traits such as shoot fresh weight and dry weight, leaf fresh weight and dry weight, root fresh weight and dry weight, number of leaves, plant height and leaf area index of each treated plant at the different time interval of 30 and 60 days after sowing (Table 2). Chilli plants pre-treated with *T*. *asperellum* + *T. harzianum* showed utmost plant growth parameters such as shoot fresh and dry weight, leaf fresh and dry weight, root fresh and dry weight, number of leaves, plant height and leaf area index followed by *T. harzianum* and *T. asperellum* (Figs. 2 and 3). The chilli seeds bio-primed with PGPF showed an increase in plant growth promoting parameters. The maximum activity of plant growth promoting traits was observed in *T. asperellum* + *T. harzianum* treated plants as compared to unprimed seed, which served as control (Fig. 4).

**Table 2:** Percentage of seed germination in control and bioagents treated samples

Treatments	Number of seeds used	Seed Germination %
Control	60	73.13 ± 1.74c
*T. asperellum*	60	87.54 ± 0.54b
*T. harzianum*	60	91.47 ± 0.61a
*T. asperellum* + *T. harzianum*	60	93.53 ± 0.52a

### Histochemical assay for lignin assessment

**Fig. 5:**
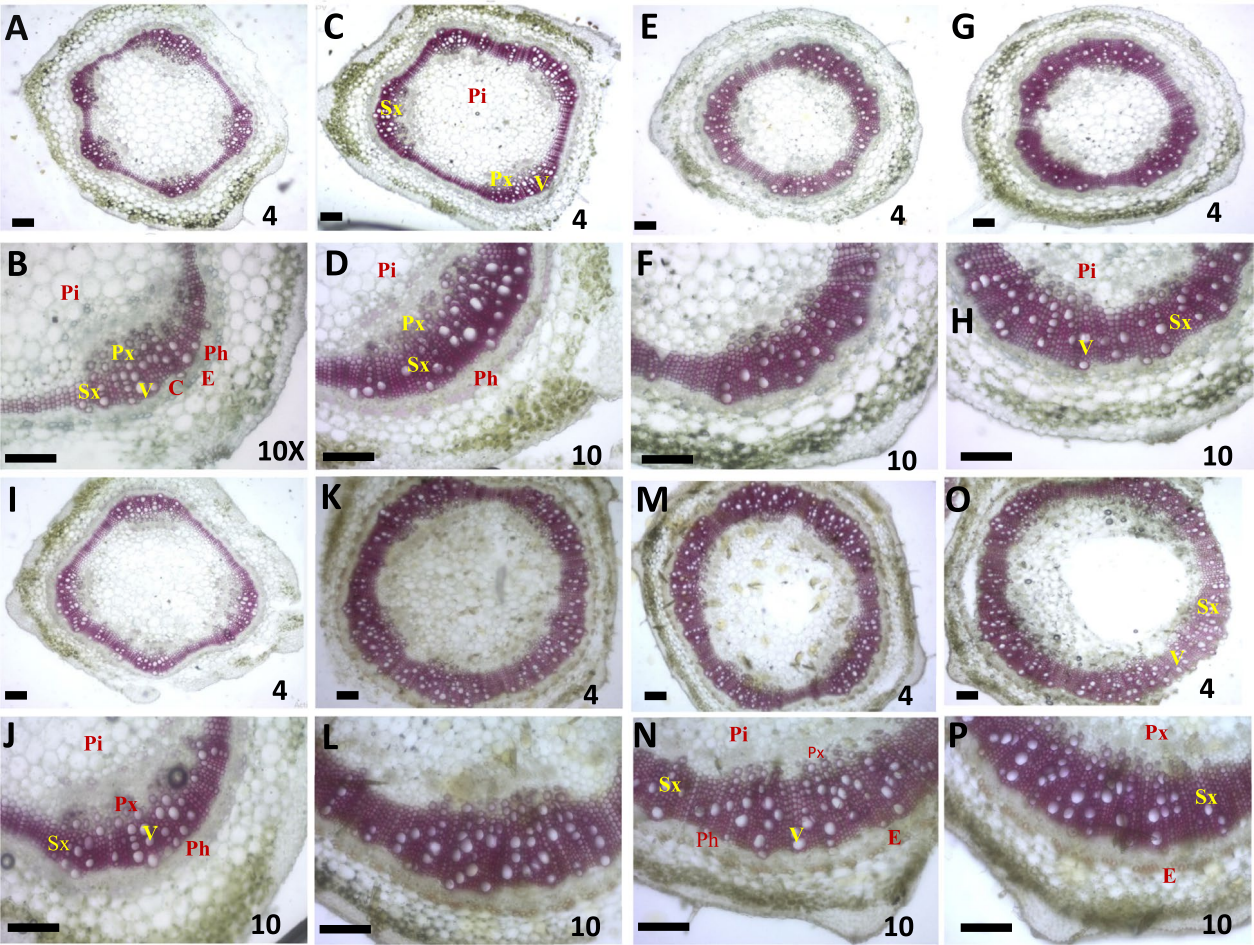
Assessment of plant mechanical barrier in the form of lignin deposition. The image indicates transverse section of chilli stem tissues with lignification in the wall of xylem strands collected from 4 different samples (unprimed, *T*. *asperellum*, *T*. *harzianum* and *T. asperellum* + *T. harzianum* bioprimed seeds) at time intervals 30 and 60 days. Intensity of red-pink color indicates the deposition of lignin. The lignified vascular bundles were seen on primary (protoxylem) and secondary xylem (metaxylem). **A-B** Microscopic view of transverse section of unprimed (control) plant samples at 30 days intervals. **C-D** Microscopic view of transverse section of *T*. *asperellum* bioprimed plant samples at 30 days. **E–F** Stem sections were taken from *T*. *harzianum* samples at 30 days. **G-H** Stem sections were taken of *T. asperellum* + *T. harzianum* samples at 30 days. **I-J** T. S of stem sections were taken from unprimed samples at 60 days. **K-L** Stem sections were taken from *T*. *asperellum* samples at 60 days. **M–N** T. S. of stem sections were taken from *T*. *harzianum* samples at 60 days. **O-P** T. S. of stem sections were taken from T. *asperellum* + *T. harzianum* samples at 60 days intervals. All the sections were observed at 4 × and 10 × magnification. Px, primary xylem; Sx, secondary xylem, Pi, pith; Ph, phloem; C, cambium; V, vessel; E, endodermis. Scale bar represents 250 µm

Seeds bioprimed with* T*. *asperellum*, *T*. *harzianum* and *T. asperellum* + *T. harzianum* showed enhanced lignin deposition as compared to unprimed and without inoculation of a pathogen (control). The lignified tissue appeared an intense coloration stained with red-pink phloroglucinol-HCl stain. The lignification of vascular tissue in untreated (control) plants had a lower quantity than bioagents treated plants. The thickness of xylem tissue as apparent through the lignin deposition was found to be greater in *T. asperellum* + *T. harzianum* treated plants followed by *T*. *harzianum*, and *T*. *asperellum* compared to unprimed and unchallenged samples. These results indicate that bioprimed seeds induced systemic resistance (ISR) against the seed-borne pathogen invasion (Fig. 5).

### Morphological study of compatible and incompatible interaction

**Fig. 6:**
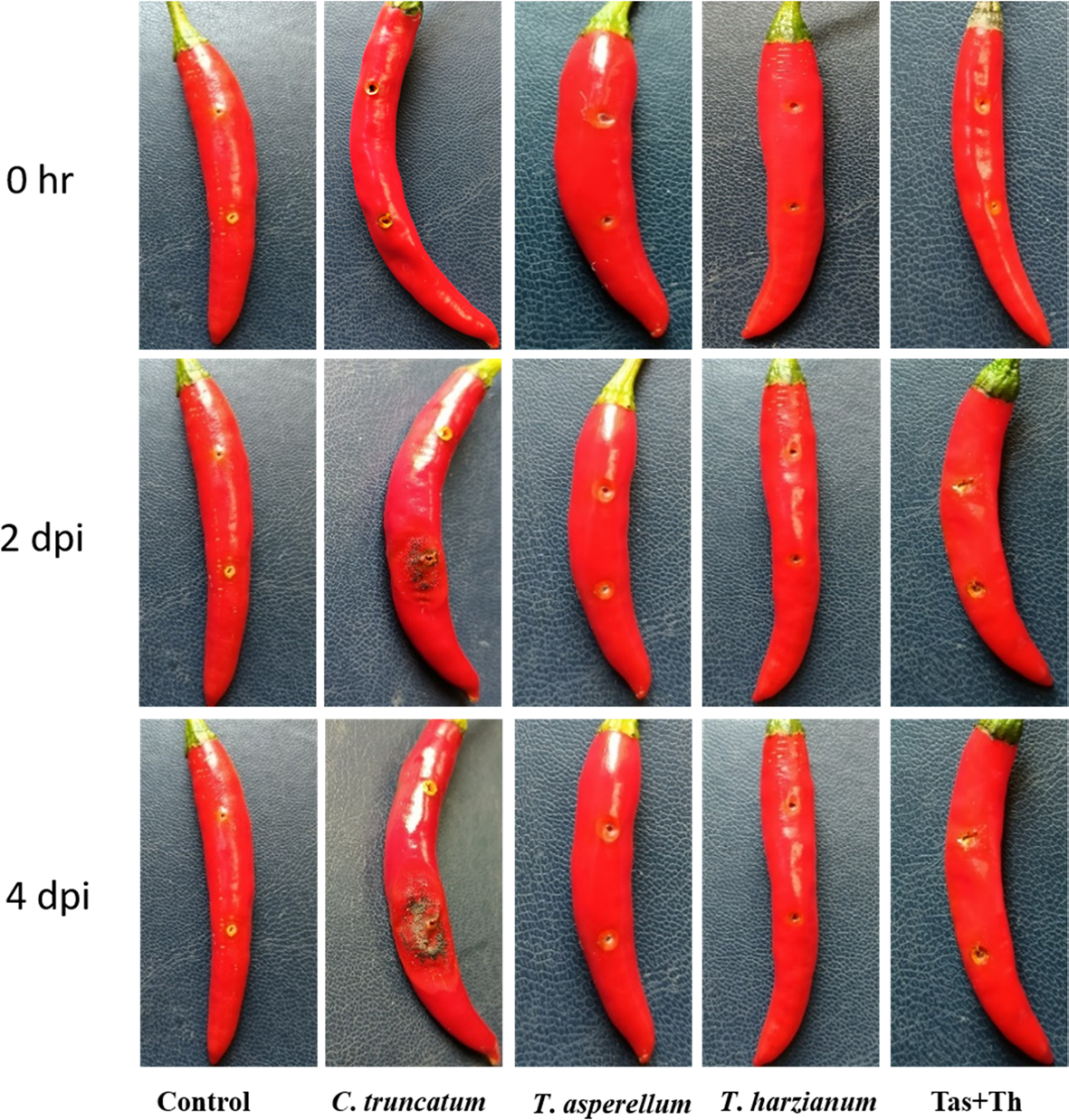
Morphological analysis of suppression of anthracnose lesion development on matured fruits of Surajmukhi variety of *Capsicum annuum* induced by *Trichoderma asperellum* and *T*. *harzianum* and in combination of treatment of *T. asperellum* + *T. harzianum* upon *C*. *truncatum* challenged condition and *C*. *truncatum* inoculated fruits. The bioprimed seeds were grown in greenhouse condition and the fruits were infected with acervuli of *C*. *truncatum* under laboratory conditions. Seeds bioprimed with *T*. *asperellum*, *T*. *harzianum* and *T. asperellum* + *T. harzianum* under challenged conditions showed no lesion compared to *C*. *truncatum* inoculated fruits. Unprimed and unchallenged (control) showed no development of anthracnose lesion. Photographs were taken at 0 h, 2- and 4-days post inoculation (dpi)

Surajmukhi (*C*. *annuum)* genotype of chilli showed the presence of typical anthracnose symptoms after 48 h post inoculation with *C*. *truncatum*. The lesions were increased progressively and the whole fruit become infected with anthracnose symptoms. The lesion length of Surajmukhi infected with *C*. *truncatum* varied from 2.5 ± 0.2 cm to 3.2 ± 0.5 cm. All the ten fruits challenged with a pathogen showed 73.20 to 77.44% PDS (percent disease score) classified as highly susceptible (Fig. 6). In comparison with fruit, the seeds primed with bioagents upon pathogen inoculated samples showed insignificant lesions that varied from 0.1 ± 0.3 cm to 0.2 ± 0.1 cm after 4 dpi of *C*. *truncatum*. The data taken from both fruit i.e., *C*. *truncatum* infected and biocontrol treated under pathogen challenged conditions display that compatible and incompatible interaction began as anticipated in chilli.

### Gene-expression studies

**Fig. 7:**
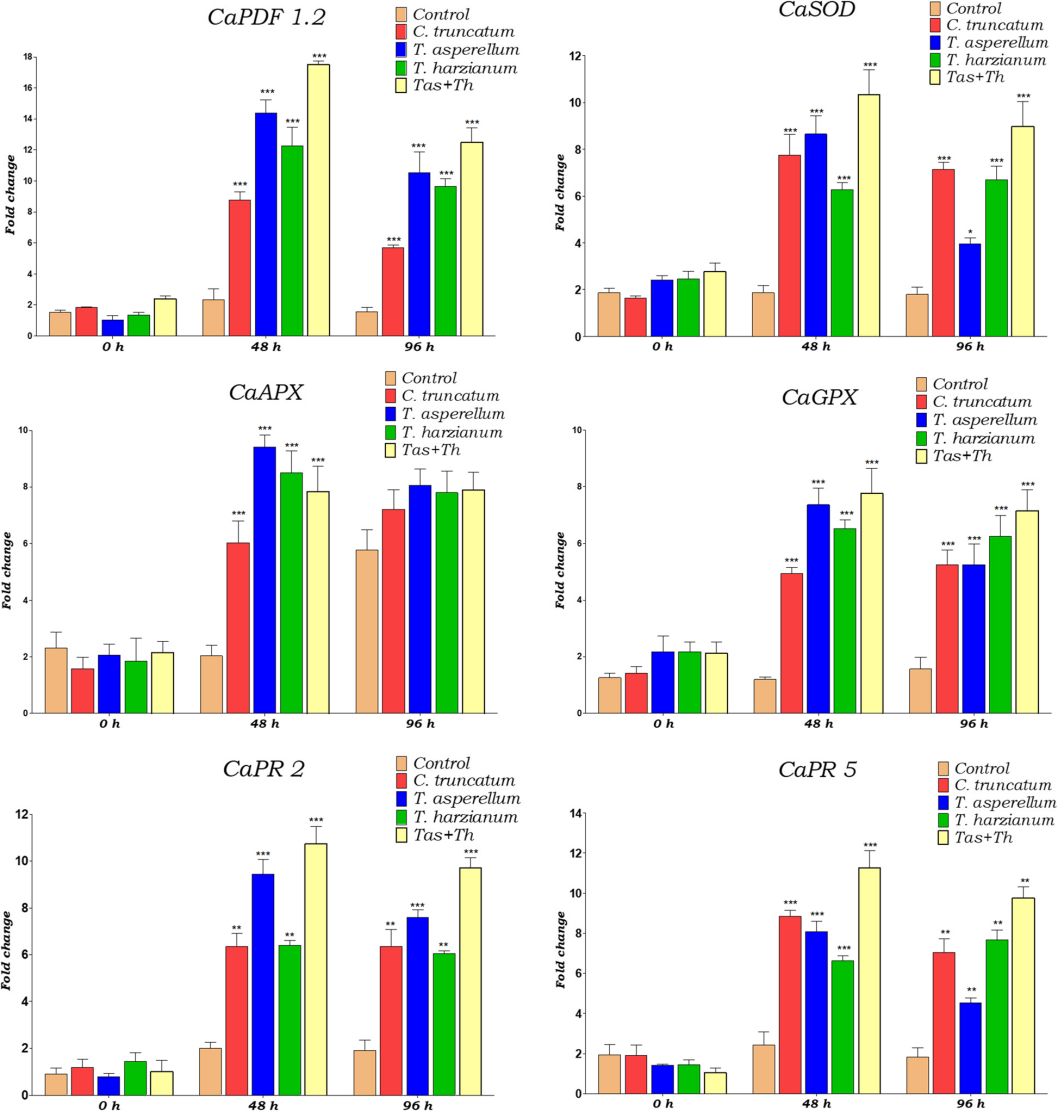
The expression of plant defensin (*CaPDF1.2*), antioxidative (*SOD*, *APx*, and *GPx*) and pathogenesis related protein (*PR-2 and PR-5*) genes in pepper bioprimed with *T*. *asperellum*, *T*. *harzianum* and *T. asperellum* + *T. harzianum* upon challenged, *C*. *truncatum* inoculated and control samples. Each data indicated the mean of triplicates value, and the vertical bars give out the same alphabetical letters are not significantly different (*p* ≤ 0.05) using Duncan’s multiple range test. The Error bars indicate SD, and the asterisk indicates a significant difference. (Student’s t-test; ⁎ *p* < 0.005; ⁎⁎ *p* < 0.01; ⁎⁎⁎ *p* < 0.001)

The time-related expression of six genes were examined in Surajmukhi genotype while compatible and incompatible interaction upon pathogen challenged and seed primed with *T*. *asperellum*, *T. harzianum* and *T. asperellum* + *T. harzianum* upon pathogen inoculated samples. The results were expressed in fold change in gene expression in pepper infected with *C. truncatum* and seed primed with *T*. *asperellum*, *T. harzianum* and *T. asperellum* + *T. harzianum* upon pathogen inoculated condition compared with unprimed (control) samples at each time interval shown in (Fig. 7).

### Differential expression of plant defensin 1.2* (CaPDF 1.2)*

The expression of *CaPDF 1.2* in pepper after the seed treated with bioagents upon challenged condition was assessed in this study. The results exhibited a substantial expression of *CaPDF1.2* under compatible and incompatible chilli-*C. truncatum* interactions. Chilli seed primed with *T. asperellum* + *T. harzianum*, *CaPDF1.2* was overexpressed at 2dpi (17.51fold) which was maximum followed by *T*. *asperellum* (14.36fold) and *T*. *harzianum* (12.24fold) upon pathogen inoculated samples. The transcript of *CaPDF1.2* in *C. truncatum* inoculated sample was significantly higher compared to unprimed (control) samples. Subsequently, the expression of *CaPDF1.2 in T. harzianum* treated sample slightly declined from 2 to 4 dpi that remarkably still higher than *C*. *truncatum* challenged sample (Fig. 7).

### Differential expression of antioxidative genes (*SOD, APx* and* GPx*)

The expression of *SOD, APx* and *GPx* genes in pepper after the seed primed with biocontrol agents upon pathogen challenged condition were examined in this study. Quantitative real time-PCR results revealed that *SOD, APx* and *GPx* were constitutively induced from 0 h to 2 dpi and decreased subsequently thereafter till 4 dpi in *C. truncatum* infected and bioagents treated samples upon *C*. *truncatum* challenged samples. The transcript of *SOD* gene was significantly up-regulated in bioprimed seeds under pathogen challenged conditions at 2 dpi. The expression of *SOD* transcript was highest in *T. asperellum* + *T. harzianum* primed seeds (10.32fold), followed by *T*. *asperellum* (8.64fold), pathogen inoculated (7.76fold), *T*. *harzianum* upon challenged (6.27fold) and compared to unprimed and unchallenged samples (1.86fold) at 2 dpi. Similarly, the constitutive expression of the *APx* gene was maximum at 2 dpi and decrease subsequently up to 4 dpi. The product of the *APx* gene has a greater affinity for binding with H_2_O_2_. It scavenges the hydrogen peroxide produced in chilli pepper against the attack of *C*. *truncatum*. The transcript level of *APx* was higher in *T*. *asperellum* (9.41fold), followed by *T*. *harzianum* bioprimed seeds (8.50fold), *T. asperellum* + *T. harzianum* (7.84fold) and upon *C. truncatum* inoculated and pathogen challenged samples (6.03fold) at 4 dpi. The expression of the *APx* gene was lowest in unprimed (control) samples (2.03fold). Likewise, the *GPx* was significantly up-regulated in bioprimed seeds under pathogen challenged at 4 dpi. The expression of *GPx* was increased from 0 to 4 dpi in *T. asperellum* compared to *T. harzianum* primed seeds upon challenged samples. The transcript level of *GPx* was maximum in *T. asperellum* + *T. harzianum* primed seed (7.7fold), followed by *T*. *asperellum* (7.34fold) and *T*. *harzianum* (6.52fold) upon pathogen inoculated and *C*. *truncatum* challenged (4.93fold) compared to control samples (1.19fold) at 2 dpi (Fig. 7).

### Differential expression of *PR* genes

Two pathogenesis related genes (*PR-2* and *PR-5*) in chilli pepper were expressed in bioprimed seeds upon pathogen challenged and unprimed (control) seeds. Results exhibited that both the *PR* genes were significantly expressed in bioprimed seeds upon pathogen challenged conditions. Transcript of *PR-2* was significantly higher in *T. asperellum* + *T. harzianum* (10.74fold), followed by *T*. *asperellum* (9.43fold) primed seeds, *C*. *truncatum* inoculated samples (6.34 fold) and *T*. *harzianum* (6.14fold) under challenged and at 2 dpi throughout the incompatible interaction in pepper upon challenged condition. A significant difference in *PR-2* gene expression was observed in pathogen challenged compared to control samples. Likewise, the transcript level of *PR-5* was maximum at 2 dpi in bioprimed seed compared to challenged and untreated and unchallenged samples. The expression level of *PR-5* was highest in *T. asperellum* + *T. harzianum*, (11.27fold) followed by pathogen challenged (8.85fold) *T*. *asperellum* bioprimed seed (8.08fold), *T*. *harzianum* (6.62fold), upon *C*. *truncatum* challenged and compared to unprimed and unchallenged samples (2.42fold) at 2 dpi and decrease subsequently to 4 dpi. Results revealed a significant difference in the transcript level of *PR-5* gene in *T*. *asperellum* primed seeds upon *C*. *truncatum* inoculation compared to *C*. *truncatum* infected samples (Fig. 7).

## Discussion

The plants suffered from different biotic and abiotic stresses under natural conditions. To combat these stresses, plants develop morphological plasticity and mechanical strength governed by plants genes [32]. The coordination of antagonistic and synergistic signaling pathways initiates the plant defense system against abiotic and biotic stresses [33]. Bioprimed seed and phyllospheric application of bioagents induced the resistance in plants by inducing the defense responsive genes and pathogenesis related (PR) protein through the activation of JA, ethylene and SA signaling pathways [34]. In this study, *T. asperellum* and *T. harzianum* isolates were evaluated for inducing the expression of six plant defense related genes that were possibly involved in the establishment of resistance in pepper against anthracnose. *C*. *truncatum* is a devastating phytopathogen causing anthracnose in the pepper of *C. annuum.* Overall, this study is the first report on the molecular defense responses in chilli pepper bioprimed with *T*. *asperellum*, *T*. *harzianum*, and *T. asperellum* + *T. harzianum* upon *C*. *truncatum* challenged condition.

*Trichoderma* spp. are universal bioagents in various ecosystems and they have an ability to colonize with roots through plant-*Trichoderma* interaction system that induced resistance in plants against several phytopathogens and enhance the plant growth that led to the development of systemic resistance. For their beneficial effect on plants, *Trichoderma* spp. have been universally used as plant protectants and productive biopesticides [25]. Several studies reported that *T. asperellum, T*. *harzianum* T-22 and *T. harzianum* enhanced the defense system in maize, wheat and cucumber plants via the development of root-*Trichoderma* interaction system against *Fusarium verticillioides* and *Ustilago maydis, F. culmorum* and *F. oxysporum* causing crown rot and wilt disease [35–37]. The foliar and bioprimed seeds with *T*. *harzianum* act as an effective elicitor of plant defense response against air and soilborne plant pathogens [25]. Bioprimed seeds with *T. asperellum* (TRU-14) and *T*. *harzianum* T274 induce resistance in *Eleusine coracana* against leaf blast disease caused by *Magnaporthe grisea* and *Phaseolus vulgaris* [38, 39]. Our study also concurs with the above findings as seeds were primed with *T*. *asperellum*, *T*. *harzianum* and *T. asperellum* + *T. harzianum* revealed positive interaction between roots and *Trichoderma* spp.

The positive interaction between root and soil microflora can promote the growth of the root, which successively promotes the growth of the shoot system [40]. Wang et al. [41] reported that *T. asperellum* 6S-2 promote the growth of apple and reduced the abundance of soil-borne pathogen, *Fusarium*. Also, seeds treated with *T*. *harzianum* Th62 significantly increased the plant height, diameter of the stem, number of branches, dry weight of root, stem, leaf, and flower in *Celosia cristata* against soil-borne pathogens*, Alternaria alternata*, *Rhizoctonia solani*, *Cystospora chrysosperma, Sclerotinia sclerotiorum* and *F. oxysporum* [42]. *T*. *koningiopsis* PSU3-2 is a potent antagonist against anthracnose in chilli pepper, caused by *C*. *gloeosporioides* [43]. *Trichoderma* spp. restricts the invasion, penetration and proliferation of fungal phytopathogens by emitting volatile compounds (antibiosis), competing for nutrient utilization and mycoparasitism [44]. Our results were corroborated with the above findings in terms of the plant growth parameters, plant height, fresh and dry weight of root and shoot, leaf area index, number of leaves, leaf fresh and dry weight, and stem diameter.

In plants, the phenylpropanoid pathway led to the synthesis of lignin, and serves as the starting point required for the production of flavonoids, coumarins and lignans [45, 46]. Phloroglucinol-HCl staining (pink or red) is the most commonly used stain for lignin determination and it is not true for stain as lignin reacts only with cinnamaldehyde end-groups of lignin to give a red-pink [47]. Phloroglucinol-HCl reacts with cinnamaldehyde yields a red-pink color in the xylem strands (primary and secondary xylem) and interfascicular fibres where these end-groups are present in lignin. Estimation of lignin deposition via phloroglucinol staining mainly stains the vascular tissue (metaxylem) not the ground tissue (sclerenchymatous) [48]. Moreover, some studies reported that *T*. *erinaceum*, *T*. *asperellum*, and *T*. *harzianum* enhanced the deposition of lignin in tomato and chilli tissues [21, 49]. Similarly in our study, enhanced lignin deposition was observed at different time intervals such as 30 and 60 days of bioprimed seeds upon *C*. *truncatum* inoculated and control samples. At 30 days of bioprimed seeds with *T. asperellum* + *T. harzianum* showed more thickness of lignin deposition followed by *T*. *harzianum* and *T*. *asperellum* compared to unprimed (control) samples. Similarly, at 60 days chilli seeds were primed with *T. asperellum* + *T. harzianum* showed a higher number of lignin layers compared to unprimed (control).

The molecular mechanism of defense related gene expression in chilli seeds bioprimed with *T*. *harzianum* and *T*. *asperellum* is not extensively examined. Although, plant root releases volatile compounds, chemicals and proteins that act as microbe-associated molecular patterns (MAMPs) to colonize the *Trichoderma* spp. Additionally, the host-specific receptors recognize particular chemical components, hydrolytic enzymes which act on pathogens and another plant cell wall could be involved as damage-associated molecular patterns (DAMPs) [50]. Further MAMPs and DAMPs induce the different signaling cascades that are essential for the expression of defense related genes against plant pathogens and mediated several hormonal actions in coordination with different signaling pathways [50]. Moreover, plants have elevated an abundance of defense related approaches to combat phytopathogenic challenges. In general, during an incompatible interaction between plant-pathogen, plants try to protect the tissue by activation of hypersensitive response, synthesis of phytoalexins and PR-proteins, deposition of lignin on the vascular strand and inducing the expression of defense related genes [51, 52]. In many cases, plants also produce peroxidase, which has a major role in defense response via phenolic cross-linking, lignification and hypersensitive response [53].

The current study highlights the significant production in the amount of defense responsive proteins accumulation in bioprimed seeds under pathogen challenged conditions as compared to compatible interactions between pathogen and chilli. Further, the study also revealed that the rhizospheric antagonists balance the defense response in plants by reducing the load of pathogen inocula and damage of tissue while enhancing the growth and development by dissolving the nutrients, production of auxin, secondary metabolites and volatile compounds [54–56]. The volatile substance produces by rhizospheric microbes suppresses the growth of plant pathogens, promotes lateral root development and induces the expression of defense responsive genes in host plants by activating the signal molecules through different cascade pathways [57–59]. Hence, defense gene activation is not constitutive and the significant accumulation of transcript of defense genes in bioprimed seeds is needed to prevent the attack of the foliar and seed-borne pathogen [52].

In our study, the transcripts of six defense genes encoding *CaPDF1.2, SOD, APx*, *GPx, PR-2* and *PR-5* genes were increased remarkably at a higher level in the bioprimed seeds upon *C*. *truncatum* challenged compared to *C*. *truncatum* inoculated and control. Hitherto, as far as we know, these defense responsive genes induced by *T*. *asperellum* and *T*. *harzianum* in chilli primed seeds have not been studied to date and therefore it is important to decipher the mechanism for developing the innate immunity that confers the resistance against anthracnose infection in detail. Plant defensin 1.2 is a cysteine-rich peptide that possesses biological activities such as antifungal, antibacterial amylase inhibitory and protease inhibitory activity which induced resistance in the plant system by developing innate immunity [60, 61]. In this sense, *CaPDF 1.2* is an eminent effector of jasmonic acid signaling and remarkably induced in several plants after the attack of phytopathogens [62]. In our study, seeds were bioprimed with *T*. *asperellum*, *T*. *harzianum,* and *T. asperellum* + *T. harzianum* induced the production of *CaPDF1.2* which involved the development of resistance in chilli pepper against anthracnose by preventing the penetration and proliferation of *C. truncatum*. In our results, the expression of *CaPDF1.2* transcript was maximum in *T. asperellum* + *T. harzianum* bioprimed seeds, followed by *T*. *harzianum*, *T*. *asperellum* upon pathogen challenged and pathogen inoculated compared to control samples at 4 dpi. In the *C. truncatum* inoculated fruits, expression of *CaPDF1.2* was significantly higher due to host–pathogen incompatibility reaction at the early stage of infection compared to unprimed and unchallenged (control) fruits at 2 and 4 dpi.

Superoxide dismutase is an antioxidative enzyme that converts the univalent reduction of ROS (O_2_^−^) to H_2_O_2_ which must be catalyzed by catalase (CAT) and peroxidase (POX) [63, 64]. The upregulation of *SOD* gene scavenge ROS generation and hence higher accumulation of H_2_O_2_ leads to the activation of phenylpropanoid pathways [49]. Further, the ROS play a major role in the activation of plant defense systems via the synthesis of secondary metabolites, deposition of lignin on the xylem strand, cell wall fortification, induction of defense related genes, and synthesis of signal molecule (SA and JA) that develop systemic acquired resistance (SAR) against the targeted phytopathogens [65, 66]. Added to this ROS molecules produced in plants during hypersensitive response led to the synthesis of several antioxidative enzymes like SOD, APx and GPx [66]. Recently Yadav et al. [2] reported the significant production of SOD in bioprimed chilli seeds upon pathogen challenged condition, *C*. *truncatum* inoculated fruit compared to unprimed and unchallenged (control). In line with our earlier findings, results indicated the expression of *SOD* transcripts in fruit tissues was more compared to leaf tissues after 4 dpi of *C*. *truncatum.* Further, *T*. *asperellum* treated seeds after 4 days inoculation of *C. truncatum* showed the highest expression of *SOD* gene compared to the control. Comparatively, fruits inoculated with the pathogen had more expression of *SOD* gene compared to *T*. *asperellum* and *T*. *harzianum* bioprimed seeds upon pathogen challenged condition at 2 dpi. Moreover, fruits of bioprimed seeds upon *C*. *truncatum* challenged and *C*. *truncatum* inoculated samples maintained a constitutive expression of the *SOD* gene compared to unprimed and unchallenged (control) samples. Therefore, we conclude that root colonization with bioagents induced the expression of the *SOD* gene in chilli fruits. Further, our findings revealed that the seeds bioprimed with *T*. *asperellum*, *T*. *harzianum* and *T*. *asperellum* + *T*. *harzianum* under pathogen challenged conditions and pathogen infected samples enhanced the expression of the *APx* gene from 0 h to 2 dpi and decreased subsequently to 4 dpi. In this sense, expression of *APx* gene was highest in *T*. *asperellum* + *T*. *harzianum* compared to *T*. *harzianum*, and *T*. *asperellum* bioprimed seeds and pathogen infected samples. A similar situation had been observed with the expression of *GPx* gene in bioprimed seeds under the challenged condition at 2 dpi and 4 dpi compared to unprimed (control), however, in this case, higher expression of the antioxidative gene was observed in *T*. *asperellum* + *T*. *harzianum* treated samples under challenged condition.

The expression of PR proteins in the plant system occurs both locally and systemically in response to biotic and abiotic stresses [67]. The PR proteins belonging to *PR-1, PR-2, PR-3* and *PR-5* play a crucial role in the induction of systemic resistance against fungal pathogens in plants [67]. In our study, two *PR* genes were induced at 2 dpi of *C*. *truncatum* and bioprimed seeds upon pathogen challenged samples and slightly decreased afterwards. The constitutive expression of the transcript of both *PR-2* and *PR-5* was observed in bioprimed seeds under *C*. *truncatum* challenged samples and *C*. *truncatum* inoculated samples. These results would imply that a basal level expression of *PR* transcript is essential for the development of immunity in the host plant against pathogenic elicitors at the early stage of infection. The transcript of both *PR-2* and *PR-5* were expressed identically in bioprimed seeds under pathogen challenged and pathogen inoculated samples at different meantime. This result coincides with Chun and Chandrasekaran [68], where, enhanced accumulation of PR-1, PR-2 (β-1,3-glucanase) PR-8 (Chitinase), and PR-10 was observed on activation of defense related genes that induced the resistance in tomato against *Fusarium andiyazi* 12,032. In the regard, a previous study demonstrates the constitutive and significant expression of PR-2 protein in the *Nicotiana tabacum* enhanced the resistance against *Rhizoctonia solani*, *Phytophthora nicotianae* and *Peronospora hyoscyami* f.sp *tabacina* [69]. However, in our study, expression of PR-2 protein was higher in *T*. *harzianum* bioprimed seeds followed by *T*. *asperellum* + *T*. *harzianum* and *T*. *asperellum* upon pathogen challenged condition, *C*. *truncatum* infected compared to unprimed (control) samples at 2 dpi. In contrast, Rout et al. [70] reported a significant accumulation of PR-5 protein in *Arabidopsis* and hot pepper after treatment with *Botrytis cinerea* and *Phytopthora capsici*. On the other hand, the accumulation of PR-5 protein was approximately similar in both the *T*. *asperellum* bioprimed seeds and pathogen inoculated samples compared to *T*. *harzianum* treated samples at 2 dpi. The above results revealed that PR-5 accumulation in *T*. *harzianum* bioprimed seeds was higher compared to *T*. *asperellum* and pathogen inoculated samples at 4 dpi. Similarly, our results of this study were also promising in the sense that colonization of *Trichoderma* spp. promoted the various plant growth parameters, strengthening the physical barriers and expression of six defense responsive genes such as *CaPDF1.2*, *SOD*, *APx*, *GPx*, *PR-2*, and *PR-5* indicating the presence of chilli-*C*. *truncatum* interactions.

## Conclusions

The present study concludes that rhizospheric and phyllospheric application of *T*. *asperellum* and *T*. *harzianum* in combination enhance the plant growth promoting trait, plant height, fresh and dry weight of root and shoot, leaf area index, number of leaves, leaf fresh and dry weight, and stem diameter. Further, the as seeds inoculated with *T*. *asperellum*, *T*. *harzianum* and in combination with treatment of *T*. *asperellum* + *T*. *harzianum* induced the strengthening of the cell wall by lignification and expression of six defense related genes *CaPDF1.2*, *SOD*, *APx*, *GPx*, *PR-2*, and *PR-5* in pepper against *C*. *truncatum*. This approach, in turn, will help to tackle the disease management problem through biopriming with *T*. *asperellum*, *T*. *harzianum* and *T*. *asperellum* + *T*. *harzianum*. Seeds inoculated with bioagents possess enormous potential to promote plant growth, modulate the physical barrier and induced the defense related genes in chilli pepper against anthracnose.

## Data Availability

The DNA sequence data generated and/or analysed during the current study are available in the GenBank nucleotide database (https://www.ncbi.nlm.nih.gov/) under accession numbers MW541903, NM_123809.4, XM_016717949.1, XM_016684466.1, XM_016717947.1, XM_016689727.1 and XM_016716165.1. The other datasets used or analyzed during the current study are available from the corresponding author upon reasonable request.
